# Structural and Enzymatic Characterization of the Choline Kinase LicA from *Streptococcus pneumoniae*

**DOI:** 10.1371/journal.pone.0120467

**Published:** 2015-03-17

**Authors:** Lei Wang, Yong-Liang Jiang, Jing-Ren Zhang, Cong-Zhao Zhou, Yuxing Chen

**Affiliations:** 1 Hefei National Laboratory for Physical Sciences at the Microscale and School of Life Sciences, University of Science and Technology of China, Hefei, Anhui, 230026, People’s Republic of China; 2 Center for Infectious Disease Research, School of Medicine, Tsinghua University, Beijing, 100084, People’s Republic of China; University of Luebeck, GERMANY

## Abstract

LicA plays a key role in the cell-wall phosphorylcholine biosynthesis of *Streptococcus pneumonia*. Here we determined the crystal structures of apo-form LicA at 1.94 Å and two complex forms LicA-choline and LicA-AMP-MES, at 2.01 and 1.45 Å resolution, respectively. The overall structure adopts a canonical protein kinase-like fold, with the active site located in the crevice of the N- and C- terminal domains. The three structures present distinct poses of the active site, which undergoes an open-closed-open conformational change upon substrate binding and product release. The structure analyses combined with mutageneses and enzymatic assays enabled us to figure out the key residues for the choline kinase activity of LicA. In addition, structural comparison revealed the loop between helices α7 and α8 might modulate the substrate specificity and catalytic activity. These findings shed light on the structure and mechanism of the prokaryotic choline kinase LicA, and might direct the rational design of novel anti-pneumococcal drugs.

## Introduction

The Gram-positive pathogen *Streptococcus pneumoniae*, is a major cause of human pneumonia, meningitis, bacteremia, otitis media and sinusitis [1, 2]. The cell wall of *S*. *pneumoniae* is essential for the bacterial survival and infection. The major components of pneumococcal cell wall are the teichoic acid and lipoteichoic acid to which abundant choline is attached in the form of phosphorylcholine [3–5]. It has been proved that lack of choline or its replacement with ethanolamine in pneumococci led to the inhibition of bacterial autolysis, the attenuation of genetic transformation and the formation of long chains [6, 7]. Moreover, recent studies have suggested that pneumococcal phosphorylcholine can interact with the host cell by binding to the platelet-activating factor receptor and the C-reactive protein [8]. In addition, a series of choline-binding proteins (CBPs), which are non-covalent linked to the phosphorylcholine of cell wall, are involved in bacterial growth, cell division and pathogenesis. For example, N-acetylmuramoyl-L-alanine amidase LytA, a major autolysin of cell wall, is important for the cell division and virulence factor release [9], whereas the major surface adhesion choline-binding protein A (CbpA) enables the pneumococcal resistance to host defense by binding to human complement factor H [10].

A gene operon termed *lic* (lipo-polysaccharide core) has been identified for the phosphorylcholine metabolism pathway of *S*. *pneumoniae* [11]. It contains two transcriptional units *lic1* and *lic2*, in which *lic1* consists of five genes *licA*, *licB*, *licC*, *tarI*, and *tarJ*, whereas *lic2* contains three genes *licD1*, *licD2* and *tacF*. The encoded proteins govern the phosphorylcholine metabolism, in which the choline transmembrane transporter LicB firstly acquires choline from external environment. Afterwards, the choline kinase LicA phosphorylates choline in the cytoplasm to form phosphocholine [12], which is further activated into CDP-choline by the phosphocholine cytidylyl transferase LicC [13]. Two phosphocholine transferases LicD1 and LicD2 catalyze the transfer of phosphocholine moiety from CDP-choline to the precursor of teichoic acid, which is synthesized by the cytidylyl transferase TarI and alcohol dehydrogenase TarJ [14]. The mature teichoic acid is flipped across the cytoplasmic membrane by the transmembrane teichoic acid flippase TacF and finally integrated into the cell wall [15]. Therefore, the choline kinase LicA is crucial due to its role in initiating phosphorylcholine metabolism pathway [16].

The choline kinases (*EC 2*.*7*.*1*.*32*) exist in almost all species [17]. To date, several crystal structures of choline kinases have been solved, such as *Caenorhabditis elegans* CKA-2 (PDB 1NW1) [17], *Homo sapiens* hCKα2 (PDB 2CKO) [18], *Plasmodium falciparum* PF14_0020 (PDB 3FI8) [19], *Plasmodium knowlesi* PKH_134520 (PDB 3C5I) [20], *Cryptosporidium parvum* CGD3_2030 (PDB 3MES) [21] and *Mesorhizobium loti* NP_106042.1 (PDB 3DXQ) [22]. They all share a similar overall structure and a conserved catalytic core. The kinetic characterization and complex structures of human hCKα2 suggest a two-step double-displacement mechanism [18, 23, 24]. A conserved residue Asp306 stabilizes the phospho-enzyme intermediate, followed by the transfer of the phosphate to choline to produce phosphocholine [24]. However, the structure and catalytic mechanism of prokaryotic choline kinases remain unknown.

Here we determined the crystal structures of apo-form LicA at 1.94 Å (apo-LicA) and two complex forms with choline (LicA-choline) and AMP/MES (LicA-AMP-MES) at 2.01 Å and 1.45 Å, respectively. Three structures presented the snapshots of the conformational change in the active site upon substrate binding and products release. Structural analysis combined with mutageneses and enzymatic assays enabled us to assign the key residues for the choline kinase activity of LicA. Structural comparison of LicA with its human homolog revealed that insertion or deletion of an active-site loop differs the activity of eukaryotic and prokaryotic choline kinases. These findings provide insights into the catalysis of prokaryotic choline kinases, and also might direct the rational design of new anti-pneumococcal drugs.

## Materials and Methods

### Overexpression and purification of LicA and mutants

The gene encoding the 289-residue LicA of *S*. *pneumoniae* R6 was initially cloned into the pET28a expression vector (Novagen) with an N-terminal 6×His tag. The recombinant plasmid was transformed into *E*. *coli* BL21 (DE3) cells by heat shock. The cells were grown at 37°C in LB medium containing 30 μg/mL kanamycin until OD_600nm_ reached about 0.8. Expression of proteins was induced with 0.2 mM isopropyl β-D-thiogalactopyranoside (IPTG) overnight at 16°C. The selenomethionine-substituted LicA protein (SeMet-LicA) was expressed in M9 minimal medium supplemented with 25 mg/L selenomethionine and other essential amino acids at 50 mg/L. Cells were harvested by centrifugation and resuspended in a lysis buffer (20 mM Tris-HCl, pH 7.5, and 100 mM NaCl). After sonication and centrifugation, the supernatant containing target protein was loaded onto a Ni-NTA column (GE Healthcare) and washed with the wash buffer (20 mM Tris-HCl, pH7.5, 100 mM NaCl, and 20 mM imidazole). The LicA protein was eluted with 500 mM imidazole and further loaded onto a Superdex 200 column (GE Healthcare) equilibrated with the buffer of 20 mM Tris-HCl, pH 7.5, 100 mM NaCl. Purified LicA proteins were concentrated to 30 mg/mL for crystallization and 1 mg/mL for enzymatic assays. Protein samples for enzymatic activity assays were stored at -80°C.

Site-directed mutagenesis was performed by using the QuickChange site-directed mutagenesis kit (Stratagene, La Jolla, CA) with the plasmid encoding the wild-type LicA as the template. The mutant proteins were expressed, purified and stored in the same manner as the wild-type protein.

### Crystallization, data collection and processing

Both native and SeMet-LicA proteins were concentrated to 30 mg/mL by ultrafiltration (Millipore Amicon) for crystallization. All crystals were grown at 16°C using the hanging drop vapor-diffusion method. The apo-LicA crystals were grown in a reservoir solution containing 0.1 M HEPES, pH 7.5, 1.2 M sodium citrate and 4% glycerol (v/v). The LicA-choline complex crystals were obtained by soaking the apo-LicA crystals with 10 mM choline overnight while LicA-AMP-MES complex crystals were grown in the reservoir solution of 0.1 M 2-(*N*-morpholino)ethanesulfonic acid (MES), pH 6.5, 30% polyethylene glycol 6000 (w/v) by the addition of AMP to the final concentration of 10 mM. All crystals were transferred to cryoprotectant (reservoir solution added with 30% glycerol (v/v)) and flash-cooled with liquid nitrogen. The diffraction data were collected at 100 K in a liquid nitrogen stream using beam line 17U with a Q315r CCD (ADSC, MAR research, Germany) at the Shanghai Synchrotron Radiation Facility (SSRF). The diffraction data were integrated and scaled using the program HKL2000 [25].

### Structure determination and refinement

The structure of LicA was determined by the single-wavelength anomalous diffraction (SAD) method using the SeMet-LicA crystals at a resolution of 2.6 Å. The selenium atoms were located using the SHELXD program of IPCAS [26]. The phase was calculated by OASIS [27] and further improved with the programs RESOLVE and Buccaneer [28, 29]. The model was built by Autobuild in PHENIX [30]. Afterwards, the initial model was subjected to the molecular replacement against the native data of the apo-form and two complex-forms using MOLREP [31]. All structures were refined with the program REFMAC5 from CCP4i [32] and rebuilt interactively using the program COOT [33]. The final structures were evaluated with the programs MOLPROBITY [34] and PROCHECK [35]. Data collection and refinement statistics were given in Table 1. All structure figures were prepared with the program PyMoL [36].

**Table 1:** Crystal parameters, data collection and structure refinement statistics.

Data Processing	SeMet-LicA	apo-LicA	LicA-AMP-MES	LicA-Choline
***Data collection***				
Space group	*P2*_*1*_ *2*_*1*_ *2*_*1*_	*P2*_*1*_ *2*_*1*_ *2*_*1*_	*P2*_*1*_ *2*_*1*_ *2*_*1*_	*P2*_*1*_ *2*_*1*_ *2*_*1*_
Unit cell (Å), (°)	70.00, 96.96, 97.92, 90.00	69.35, 96.47, 97.59, 90.00	62.34, 62.86, 68.75, 90.00	70.39, 96.69, 98.60, 90.00
No. of molecules per asymmetric unit	2	2	1	2
Resolution range (Å)	50.00–2.60	50.00–1.94	50.00–1.45	50.00–2.01
Unique reflections	21,083 (2,064)^a^	47,710 (4,732)	47,856 (4,723)	44,656 (4,354)
Completeness (%)	99.8 (100)	97.2 (97.8)	98.5 (98.8)	98.2 (98.0)
<I/σ(I)>	11.9 (5.9)	20.3 (2.8)	12.3 (2.0)	16.4 (3.4)
R_merge_ ^b^ (%)	16.4 (66.6)	6.2 (52.9)	8.5 (65.1)	9.4 (57.1)
Average redundancy	9.5 (9.7)	3.0 (3.0)	3.6 (3.6)	3.4 (3.4)
***Structure refinement***				
Resolution range (Å)		50.00–1.94	46.39–1.45	50.00–2.01
R-factor^c^/R-free^d^ (%)		21.6/26.2	19.3/21.4	20.0/23.7
Number of protein atoms		4,587	2,341	4,679
Number of water atoms		289	328	312
RMSD^e^ bond lengths (Å)		0.010	0.006	0.010
RMSD bond angles (°)		1.107	1.042	1.138
Mean B factors (Å^2^)		39.2	19.4	36.2
Ramachandran plot^f^ (%)				
Most favored (%)		94.8	96.8	95.8
Additional allowed (%)		5.2	3.2	4.2
Outliers (%)		0	0	0
PDB entry		4R77	4R78	4R7B

^a^The values in parentheses refer to statistics in the highest bin.

^b^R_merge_ = ∑_hkl_∑_i_|Ii(hkl)-<I(hkl)>|/∑_hkl_∑_i_I_i_(hkl), where I_i_(hkl) is the intensity of an observation and <I(hkl)> is the mean value for its unique reflection; Summations are over all reflections.

^c^R-factor = ∑_h_||Fo(h)|-|Fc(h)||/∑_h_|Fo(h)||, where |Fo| and |Fc| are the observed and calculated structure-factor amplitudes, respectively.

^d^R-free was calculated with 5% of the data excluded from the refinement.

^e^Root-mean square-deviation from ideal values.

^f^Categories were defined by Molprobity.

### Enzymatic assays

The choline kinase activity of LicA and mutants were measured by high performance liquid chromatography (HPLC) assays [23]. The reaction mixture in a final volume of 50 μL contained 20 mM Tris-HCl, pH 8.0, 100 mM NaCl, 2 mM MgCl_2_, 2 mM ATP and choline at varying concentrations. The reaction was triggered by the addition of 50 nM LicA and was lasted for 10 min at 37°C. Then the reaction was terminated by boiling at 100°C for 10 min. Each sample was centrifuged at 12,000 × g for 10 min, then 40 μL supernatant was applied to the HPLC system (Agilent 1200 Series). The buffer of 100 mM K_2_HPO_4_/KH_2_PO_4_, pH 6.5 was used as the mobile phase to equilibrate the column (Eclipse XDB-C18 column, 5 μm, 4.6 × 250 mm; Agilent) and the components were separated at a flow rate of 1 mL/min. The product ADP was monitored with the absorption at 254 nm and was assigned based on the retention time of the standards. All the measurements were done in triplicate. The final enzymatic kinetic parameters were calculated based on the yield of ADP by nonlinear fitting to the Michaelis-Menten equation with the program GraphPad Prism.

## Results and Discussion

### Overall structure

Each asymmetric unit contains two molecules of LicA. The interface between the two molecules is about 650 Å^2^, which is not large enough to maintain a stable dimer. In fact, LicA exists as a monomer in solution, which was confirmed by gel-filtration chromatography (S1 Fig.). The overall structures of two molecules are very similar with a root-mean-square deviation (RMSD) of 0.4 Å over 280 Cα atoms, thus we took molecule A for further structural analyses.

**Fig 1:**
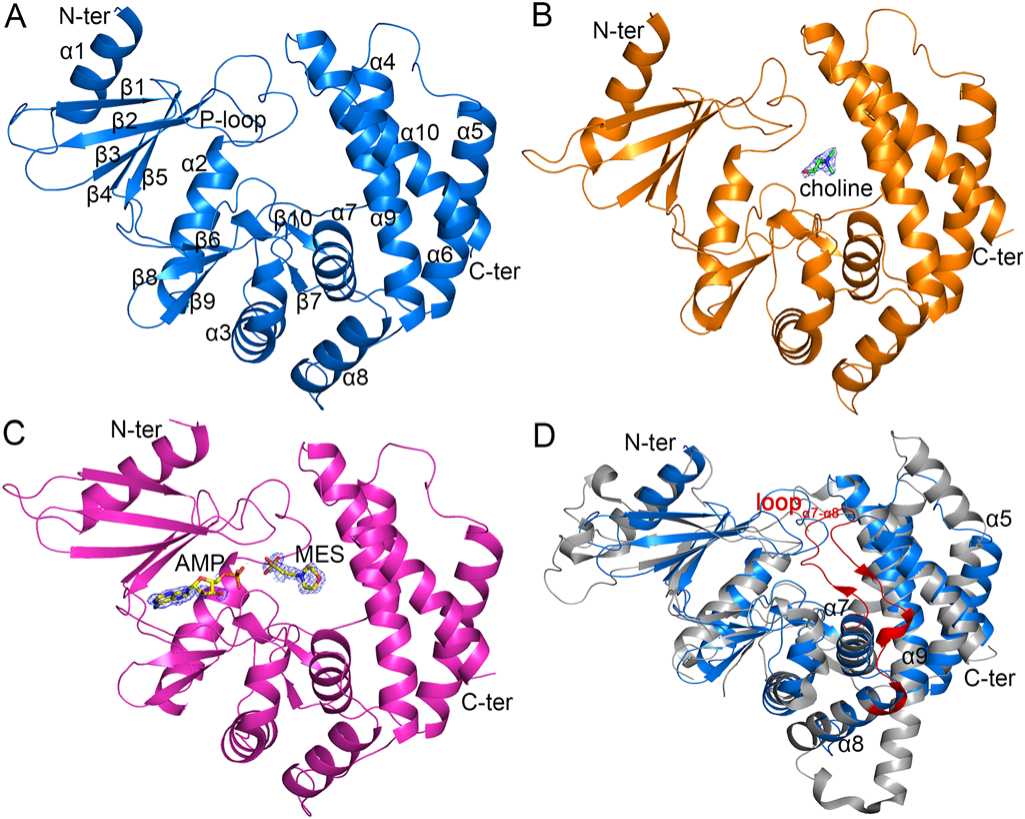
Overall structure of LicA. (A) apo-LicA (blue). The secondary structure elements are labeled sequentially. (B) LicA-choline complex structure (orange). The substrate choline is shown as green sticks, with the *Fo-Fc* electron-density omit map contoured at 3.0 sigma. (C) LicA-AMP-MES complex structure (magenta). The AMP and MES molecules are shown as yellow sticks, with the *Fo-Fc* electron-density omit map contoured at 3.0 sigma. (D) Superposition of overall structures between LicA (blue) and *H*. *sapiens* hCKα2 (gray). The loop_α7-α8_ from hCKα2 is labeled in red.

The structure of LicA adopts a protein kinase-like fold which comprises of an N-terminal domain (residues 1–92) and a C-terminal domain (residues 93–283) (Fig. 1A). The N-terminal domain contains two helices (α1 and α2), which sandwich a twisted five-strand antiparallel β-sheet (β1-β5). The C-terminal domain consists of eight α-helices (α3-α10) surrounding five short β-strands (β6-β10). The N- and C-terminal domains form a catalytic crevice that harbors several conserved motifs: the ATP-binding motif [18, 37] in the loop (the so-called P-loop, residues Gly26-Asn31) connecting β1 and β2, the phosphotransferase Brenner’s motif [(CS)HNDhX_3_N] [38] in the loop (Ser172-Asn181) between β7 and β8 and choline kinase motif [(ILV)X_2_ID(FWY)E(YF)X_3_NX_3_(FYW)DX_6_E] [17] in the region of β9, β10 and α7 (Leu190-Glu213). These motifs were reported to be indispensable for the catalysis [18].

In the complex structure of LicA-choline, a molecule of choline is located at the active site of the C-terminal domain (Fig. 1B), whereas in the complex structure of LicA-AMP-MES, an AMP molecule and an MES molecule occupy the active site (Fig. 1C). The MES molecule, which should be introduced from the crystallization buffer, occupies the corresponding position of choline in LicA-choline, whereas the AMP molecule is stabilized by the N-terminal domain. The *Fo-Fc* omit map at 3 σ revealed poor density of the ribose-phosphate moiety of AMP, as reflected by its low occupancy. The physiological substrate and product of LicA are ATP and ADP, respectively, both of which are larger than the molecule of AMP. Thus the AMP molecule could only partially occupy the active-site pocket with a relatively lower binding affinity, which was further indicated by the fewer hydrogen bonds of LicA-AMP, compared to ADP binding to hCKa2 (PDB 2CKP) [18].

Structural homology search using DALI [39] indicated that the overall structure of LicA resembles the members of the choline kinase family, although LicA only shares a sequence identity of less than 25% with the members of known structure. The closest member is the putative choline kinase NP_106042.1 from *M*. *loti* (PDB 3DXQ, Z-score = 26.4, RMSD = 3.0 Å over 268 Cα atoms) [22], whose biochemical information has not been elucidated. Other similar structures include *P*. *knowlesi* choline kinase PKH_134520 (PDB 3C5I, Z-score = 25.1, RMSD = 2.5 Å over 272 Cα atoms) [20] and *H*. *sapiens* choline kinase hCKα2 (PDB 2CKO, Z-score = 24.8, RMSD = 2.6 Å over 275 Cα atoms) [18]. Superposition of the LicA structure against that of human hCKα2 revealed that they share a similar overall structure, despite that hCKα2 contains a more flexible N-terminal loop and two longer helices (corresponding to α5 and α9 in LicA) (Fig. 1D). The most significant difference is the variations of the loop_α7-α8_ in the active site. The loop_α7-α8_ of LicA has only 5 residues, but the corresponding loop of hCKα2 possesses 21 residues, which forms a long hairpin-like structure and extends to the top of the choline-binding pocket (Fig. 1D, in red). In previous reports, this loop was proposed to modulate the access of the substrate choline [17, 18]. Thus the deletion of loop_α7-α8_ might differ the catalytic activity of LicA from that of hCKα2.

### The active site

**Fig 2:**
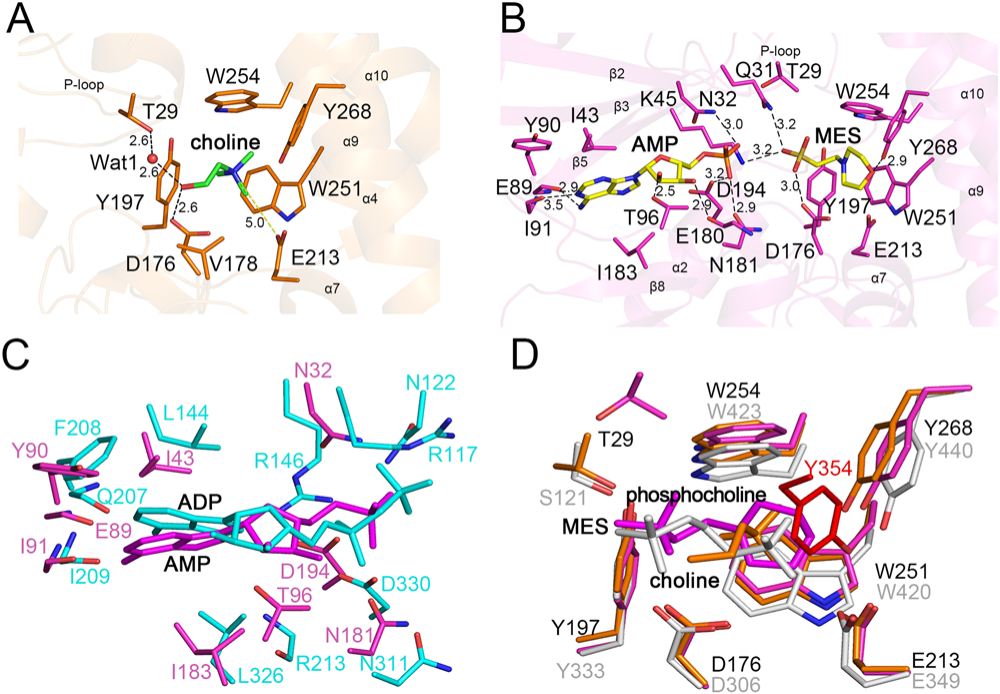
The active site. (A) The choline-binding site of LicA. The binding residues and choline are shown as orange and green sticks, respectively. The secondary structure elements are shown as semi-transparent cartoon. The hydrogen bonds and polar interactions are indicated as dashed lines. (B) The AMP- and MES-binding sites of LicA. The binding residues are shown as magenta sticks whereas the AMP and MES molecules are shown as yellow sticks. (C) Superposition between LicA-AMP-MES (magenta) and human hCKα2-ADP (cyan). (D) Structural comparison of the choline-binding sites of LicA-choline (yellow), LicA-AMP-MES (magenta) and hCKα2-phosphocholine (gray).

In the LicA-choline structure, four aromatic residues, Tyr197, Trp251, Trp254 and Tyr268 together with Val178 form a hydrophobic pocket to accommodate the quaternary amine moiety of choline (Fig. 2A). Glu213 at the rim of the pocket makes a charge charge interaction with the positively charged quaternary amine of choline. In addition, residues Thr29 (mediated by a water molecule) and Asp176 form two hydrogen bonds with the hydroxyl moiety of choline. The corresponding residues Ser121 and Asp306 in hCKα2 are proved to be essential for catalysis [18].

In the LicA-AMP-MES structure, the MES molecule presents in the same place as choline in the structure of LicA-choline (Fig. 2B). The morpholine ring of MES is stabilized by residues Tyr197, Glu213, Trp251, Trp254 and Tyr268, whereas the sulfonic acid moiety forms hydrogen bonds with Gln31, Lys45 and Asp176. The AMP molecule is located at the N-terminal domain. The adenine ring of AMP makes hydrophobic interactions with residues Ile43, Tyr90 and I183, and also forms hydrogen bonds with the main chains of Glu89 and Ile91. The ribose moiety makes hydrogen bonds with the side chain of Thr96 and the main chain of Glu180, respectively, whereas the α-phosphate moiety interacts with Asn32, Asn181 and Asp194.

Comparison between the active site of LicA-AMP-MES and that of hCKα2-ADP (PDB 2CKP) [18] showed that the AMP molecule in LicA could be well superimposed to the product ADP in hCKα2-ADP (Fig. 2C). AMP adopts a same conformation as ADP, and both of which share a similar binding pattern. However, the ribose of AMP in LicA makes hydrogen bond with side chain of Thr96, and the α-phosphate moiety interacts with Asn32, Asn181 and Asp194, whereas the ribose of ADP in hCKa2 makes hydrogen bond with the main chain of Arg213, the α-phosphate moiety interacts with Arg146, Asn311 and Asp330, and the β-phosphate moiety interacts with Arg177 and Asn122. For the choline-binding pocket (Fig. 2D), the choline or MES molecule in LicA could be well aligned to the phosphocholine in hCKα2-phosphocholine (PDB 2CKQ) [18]. The key hydrophobic residues involved in stabilizing the quaternary amine of choline or the morpholine ring of MES in LicA are structurally conserved in hCKα2-phosphocholine. However, in hCKα2, the residue Tyr354 in the long hairpin-like loop_α7-α8_ interacts with the quaternary amine of phosphocholine (Fig. 2D), but this interaction is missing in LicA due to the fact that the corresponding loop_α7-α8_ is much shorter (Fig. 1D). In addition, the phosphate moiety of phosphocholine in hCKα2 is stabilized by Ser121 and Asp306, whereas the corresponding residues Thr29 and Asp176 in LicA interact with choline. Moreover, in the structure of LicA-AMP-MES, only Asp176 stabilizes the sulfonic acid moiety of MES, with Thr29 on the P-loop flipping away from the active-site pocket. The similar binding patterns suggest that AMP and MES in LicA-AMP-MES might mimic the native products ADP and phosphocholine, respectively. On the other hand, the differences of the active site between LicA and hCKα2 revealed the species specificity which might guide the design of inhibitors against choline kinases.

### The conformational changes during catalysis

**Fig 3:**
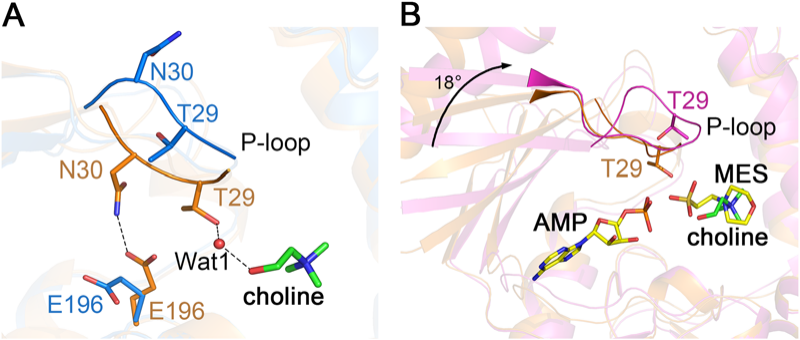
Conformational changes in the active site. (A) Comparison between apo-LicA (blue) and LicA-choline (orange) with P-loop shown as cartoon. The choline molecule and the interacting residues are shown as sticks. The hydrogen bonds are indicated as dashed lines. (B) Comparison between LicA-choline (orange) and LicA-AMP-MES (magenta). The choline molecule is shown as green sticks while AMP and MES are shown as yellow sticks. The 18° rotation of the N-terminal domain is indicated as black arrow.

Superposition of the LicA-choline complex structure against the apo-LicA structure revealed an RMSD of 0.4 Å over 278 Cα atoms indicating slight conformational changes of the overall structure. However, drastic differences were found in the active site. Upon the binding of the substrate choline, Thr29 forms a water-mediated hydrogen bond with choline, resulting in a shift of the P-loop about 4 Å towards the choline molecule. The residue Asn30 in the P-loop also moves to the active site and makes a hydrogen bond with Glu196 to further stabilize the P-loop (Fig. 3A). Therefore, the active-site pocket undergoes a change from an open to a closed conformation upon choline binding.

The human hCKα2-phosphocholine structure revealed a closed conformation with the P-loop closure and 16° rotation of the two domains, upon phosphocholine binding [18]. Similar to hCKα2, the N-terminal domain of LicA-AMP-MES rotates approximately 18° towards the C-terminal domain comparing to apo-LicA by the program DynDom [40], making N- and C-terminal domains much closer to each other. Structural comparison between the two complex structures LicA-choline and LicA-AMP-MES yields an RMSD of 1.4 Å over 273 Cα atoms. The P-loop in LicA-choline is close to the active site and the residue Thr29 of P-loop interacts with choline, whereas the P-loop in LicA-AMP-MES is far away from the active site and adopts a conformation similar to that in apo-LicA structure. In addition, the N-terminal domain in LicA-AMP-MES also shows a rotation of about 18° towards the C-terminal domain comparing to LicA-choline structure (Fig. 3B). Therefore, based on the three structures of LicA and two complex structures of hCKα2, we propose a catalytic cycle of choline kinases (S2 Fig.). In the apo-form state, the enzyme adopts an open conformation (1), with the P-loop protruding outwards from a wider active-site pocket. Upon the binding of substrate choline, the P-loop shifts towards the active site to stabilize the substrate, resulting in a closed conformation (2). Binding of the second substrate ATP triggers the catalytic reaction which might lead to domain rotation in addition to P-loop closure (3). When the products are formed, the P-loop is kicked away from the active site (4), accompanied with the release of the products (5) and turnover of the enzyme.

### Choline kinase activity of LicA

**Fig 4:**
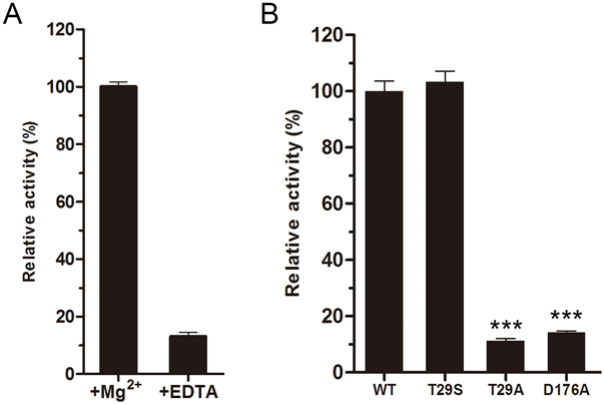
Enzymatic activities of wild-type LicA and mutants. (A) Effect of magnesium or EDTA on LicA activity. (B) The relative enzymatic activities of the wild-type LicA and mutants. One-way ANOVA with a post hoc Dunnett test is used for the comparison of statistical significance. The P values of <0.05, 0.01 and 0.001 are indicated with *, ** and ***, respectively.

The choline kinase activity was tested by HPLC assays. LicA showed an optimal activity at pH 8.0 in the presence of Mg^2+^. Despite no metal observed in the three structures, we propose that the activity of LicA is dependent on Mg^2+^, as deprivation of metal ions using ethylenediaminetetraacetic acid (EDTA) drastically diminished its activity by 6 fold (Fig. 4A). Similar case has also been found in ChoKα1, that the metal Mg^2+^ was found in the substrate-binding pocket to stabilize the phosphate group of ADP [23]. The wild-type LicA has *K*_*m*_ and *k*_*cat*_ values of 0.15 ± 0.05 mM and 5.1 ± 0.7 s^-1^, respectively, resulting in the activity (*k*_*cat*_ */K*_*m*_) of 33.3 s^-1^mM^-1^. Mutation of the conserved Asp176 to alanine decreased the relative activity to about 14% of the wild-type suggesting Asp176 is critical for catalysis (Fig. 4B). The corresponding residue Asp306 in hCKα2 makes a critical hydrogen bond with the inorganic phosphate anion to stabilize the phosphor-enzyme intermediate [24]. In addition, mutation of the choline-binding residue Thr29 to alanine decreased the relative activity to about 10% of the wild-type. However, the T29S mutant retained the full activity, supporting the importance of the hydroxyl group at this position. In addition, other conserved residues, such as Asn181, Asp194 and Glu196 are also important for catalysis, which were also found in hCKα2 [18]. Site-directed mutageneses together with enzymatic assays indicate that LicA might also adopt the two-step double-displacement mechanism [24].

**Fig 5:**
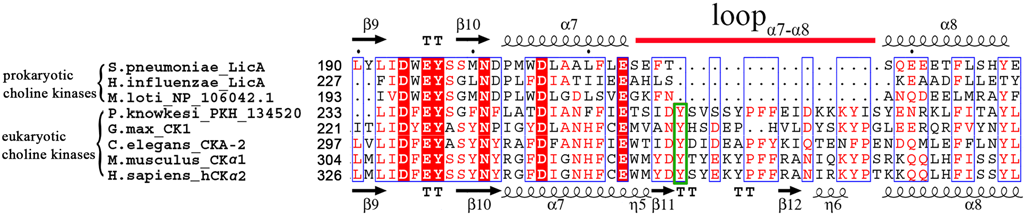
Multiple-sequence alignment of eukaryotic and prokaryotic choline kinases. The alignment is generated with the programs Multalin [46] and Espript [47]. Secondary structure elements of LicA and hCKα2 are indicated on the top and bottom of the sequences, respectively. The loop_α7-α8_ that differs the eukaryotic choline kinases from that of prokaryotes is indicted by a red line on the top of the sequence. The conserved tyrosine residue in the loop_α7-α8_ is labeled by a green box.

The human choline kinase hCKα2 has an activity (*k*_*cat*_ */K*_*m*_) of 848 s^-1^mM^-1^, which is 25-fold higher than that of LicA (33.3 s^-1^mM^-1^) [18, 41–44]. Structural comparison revealed the loop_α7-α8_ at the choline-binding pocket might attribute to the different enzymatic activities. As a prokaryotic choline kinase, LicA has a very short loop_α7-α8_ of 5 residues, resulting in a relatively open choline-binding pocket, whereas the corresponding loop in hCKα2 is about 21-residue long, which extends to active site and covers the choline-binding pocket (Fig. 1D). In addition, an additional residue Tyr354 on loop_α7-α8_ stacks the quaternary amine of phosphocholine in hCKα2 (Fig. 2D). This tyrosine residue is exclusively conserved in eukaryotic choline kinases, but it is absent in prokaryotes (Fig. 5). Thus an insertion in the loop_α7-α8_ of the eukaryotic hCKα2 contributes to the high catalytic activity, maybe via increasing the binding affinity of the substrate choline. The higher activity of eukaryotic choline kinases is also consistent with the requirement of higher amount of phosphocholine, involved in the phosphatidylcholine biosynthesis for membrane phospholipid [45]. Multiple-sequence alignment revealed that the longer loop_α7-α8_ is a common feature in eukaryotic choline kinases, but this loop is much shorter in prokaryotic choline kinases (Fig. 5). Therefore, the loop_α7-α8_ at the choline-binding pocket plays an important role in substrate specificity and catalytic activity of choline kinases, and the variation of the loop length might differ the prokaryotic choline kinases from their orthologs in eukaryotes.

## Supporting Information

S1 Fig The gel filtration data showing the monomeric state of LicA. The standard curve was inserted as an inlet.
(TIF)

S2 Fig A schematic diagram depicting the catalytic cycle of choline kinases. (TIF)
