# Supplementary figures and images for: Structural and Enzymatic Characterization of the Choline Kinase LicA from Streptococcus pneumoniae
Source: PLoS One. 2015 Mar 17;10(3):e0120467. doi: 10.1371/journal.pone.0120467 (PMC4364537; doi:10.1371/journal.pone.0120467)

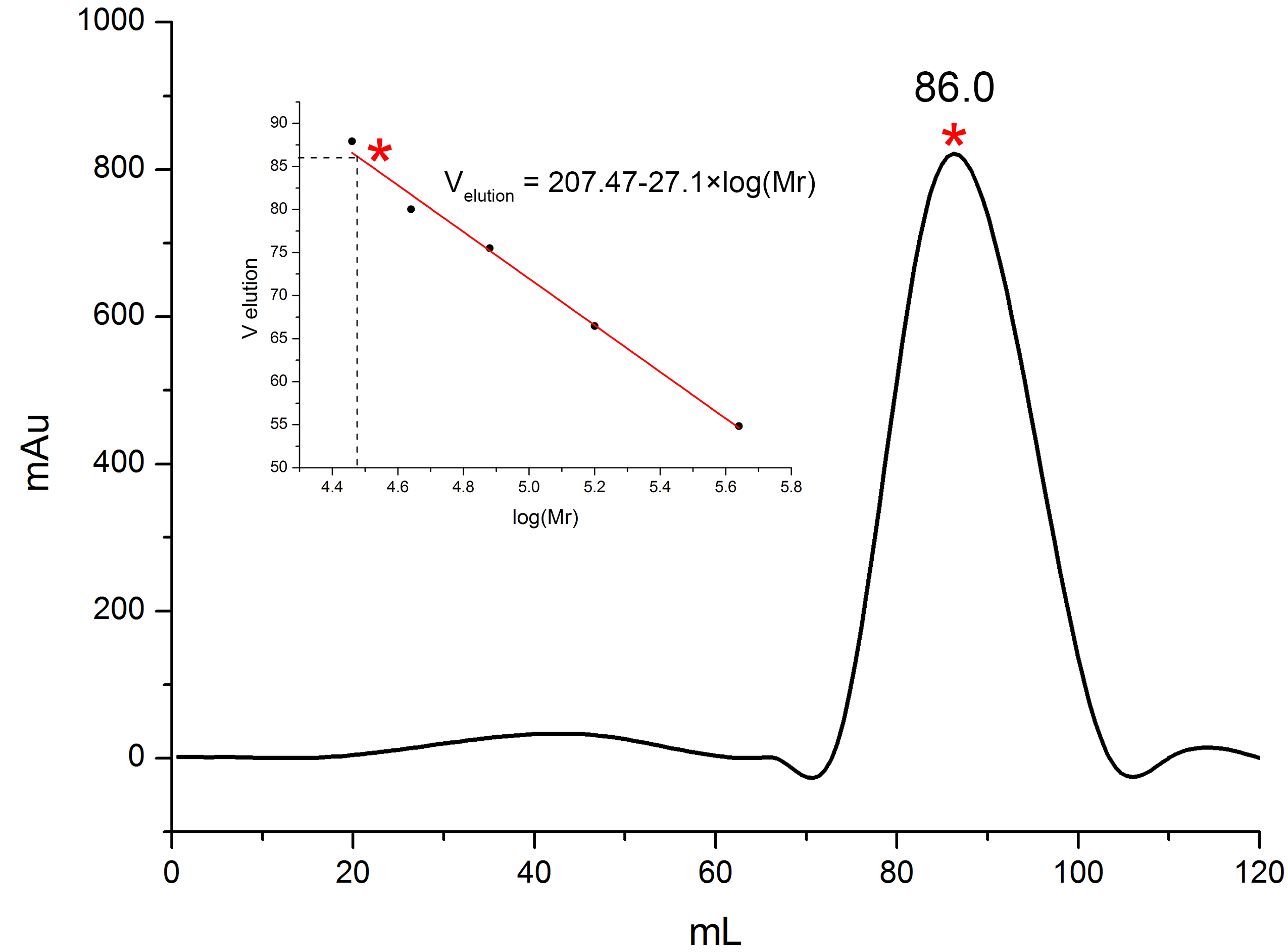

Supplement: S1 Fig — The standard curve was inserted as an inlet. (TIF) [file pone.0120467.s001.tif]

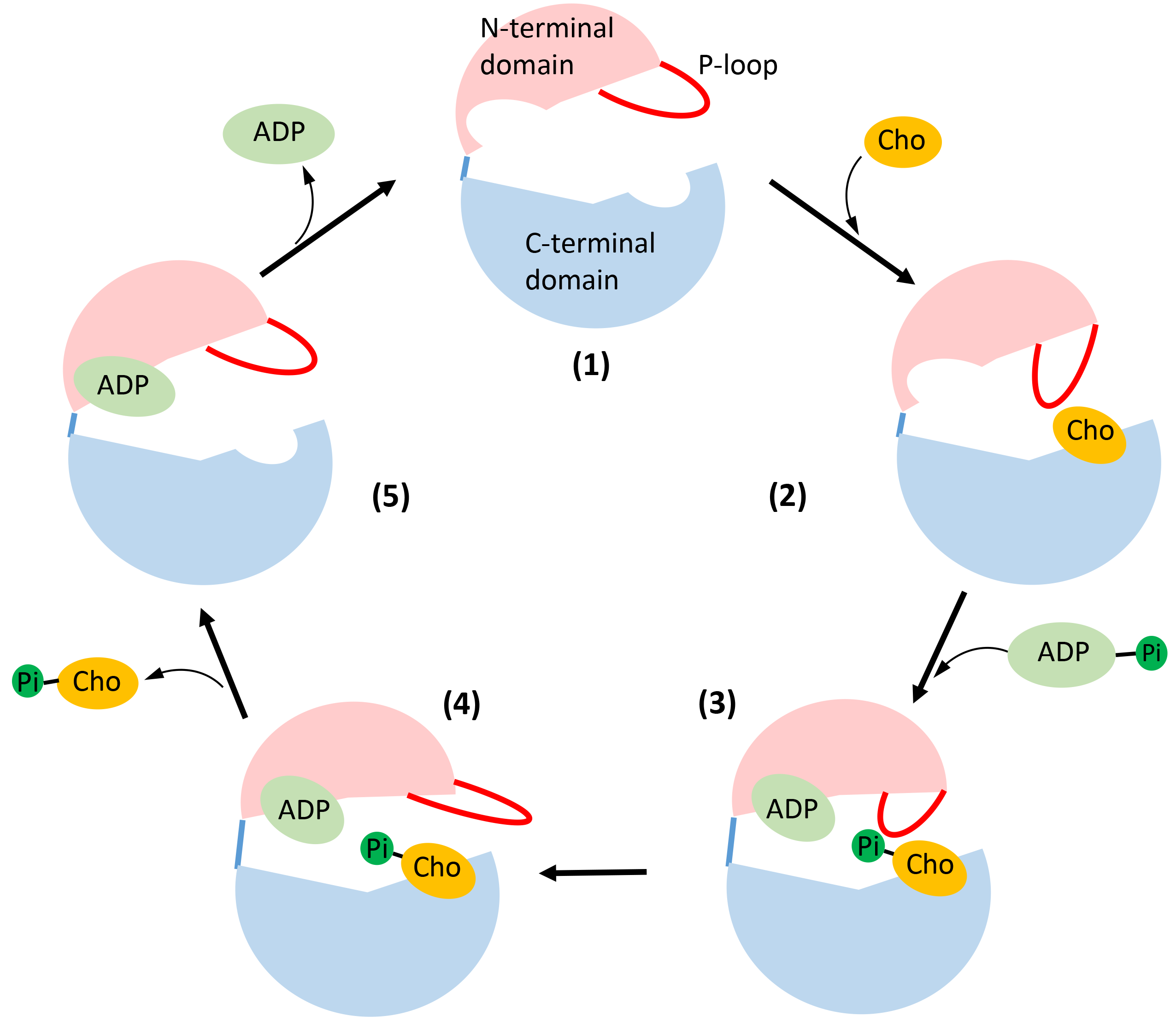

Supplement: S2 Fig — (TIF) [file pone.0120467.s002.tif]
